# Exploring the influence of pressure-induced semiconductor-to-metal transition on the physical properties of cubic perovskites FrXCl_3_ (X = Ge and Sn)

**DOI:** 10.1016/j.heliyon.2024.e27581

**Published:** 2024-03-17

**Authors:** Asif Hosen, Md. Rasidul Islam, Shahriar Haque Badhan

**Affiliations:** a Department of Materials Science and Engineering, Khulna University of Engineering & Technology (KUET), Khulna, 9203, Bangladesh; b Department of Electrical and Electronic Engineering, Bangamata Sheikh Fojilatunnesa Mujib Science & Technology University, Jamalpur, 2012, Bangladesh

**Keywords:** Perovskite, FrGeCl_3_, FrSnCl_3_, DFT, Physical properties

## Abstract

Even though lead halide perovskites have outstanding physiochemical properties and improved power conversion efficiency, most of these compounds threaten their future commercialization because of their instability and highly toxic nature. Thus, it is preferable to use stable alternative elements rather than lead to make environmentally friendly perovskite material that will have comparable optical and electronic properties to those constructed from Pb-based perovskites. However, devices constructed from lead-free perovskites typically display a lower power conversion efficiency. Applying hydrostatic pressure could be deemed an effective method to alter the physical properties of these compounds. This not only improves their performance in application but also reveals significant correlations between structure and properties. This work uses DFT to investigate the structural, electronic, optical, and elastic properties of non-toxic, francium-based halide perovskites FrXCl_3_ (X = Ge, Sn) at different levels of hydrostatic pressures that vary from 0 to 10 GPa. The estimated structural parameter's strong correlation with the data from earlier studies ensures the accuracy of the current findings. Pressure causes the Fr−Cl and Ge (Sn)–Cl bonds to shorten and become stronger. The electronic property calculations demonstrated that both compounds are direct band-gap semiconductors. The application of pressure leads to a linear reduction in the band gap (semiconducting to metallic state) and raises the electronic density of states around the Fermi level by forcing the valence band electrons upward, indicating that the optoelectronic device's performance can be tuned and improved. The values of the dielectric constant, absorptivity and reflectivity showed an increasing tendency with pressure. As the pressure applied to the compounds increases, the absorption spectra show a redshift. These findings suggested that the FrXCl_3_ (X = Ge and Sn) compound becomes more appropriate for usage in optoelectronic applications under pressure. Furthermore, our examination of the mechanical properties indicates that both FrGeCl_3_ and FrSnCl_3_ exhibit mechanically stability, and ductility. Interestingly, we observe an increase in ductility as pressure levels rise.

## 1 Introduction

The expansion of businesses all over the world has led to an unprecedented rise in the amount of energy that is used; the vast majority of this energy originates from resources that are not replenishable. Due to the accidental emission of carbon dioxide, the usage of traditional sources of energy is linked to serious problems for the surrounding environment. Solar energy, on the other hand, is by far the most plentiful form of sustainable energy. In order to fully harness the potential of this natural resource, researchers and scientists are making significant efforts towards developing efficient, effective, and environmentally friendly solar cells. The utilization of non-toxic, Pb-free halide perovskite material in the construction of solar cells poses a substantial barrier to commercialization [1]. Because of this, several theoretical and practical studies have been conducted recently where Pb was substituted by a suitable metal cation [[2], [3], [4], [5], [6]]. Solar cells made of perovskite compounds have recently been demonstrated to be competitive with traditional solar cells based on silicon, owing to their high efficiency combined with lower manufacturing costs [[7], [8], [9]]. In this regard, perovskite materials, with their versatile optical characteristics, have lately demonstrated promising results in optoelectronic applications [[10], [11], [12], [13], [14], [15], [16]].

The unique characteristics of perovskite materials, which include superconductivity, strong magnetoresistance, and increased electron mobility, among others, have attracted a lot of attention [[17], [18], [19], [20]]. They also have outstanding potential for commercial uses in fields like photovoltaics, memory devices, optical coatings, fuel cells, and lens materials [[21], [22], [23], [24]]. The typical formula for cubic perovskites is ABX_3_, with A and B standing for the cations, while X may be either halogens or oxygen, representing an anion [25,26]. Significant progress has been made in enhancing the power conversion efficiency (PCE) of solar cells utilizing perovskite material, rising from 3.8% to well over 25% in recent years [27,28]. The highest observed PCE for Pb-based perovskite (MAPbI_3_) solar cells is 25.2% [29]. Harmful and dangerous lead substances have continued to demonstrate promising results in luminescence activities [30,31]. However, the presence of humidity, moisture, temperature, and ultraviolet light present in ambient circumstances causes these materials to be unstable [32]. Another concern lies in the use of lead (Pb), a toxic material that poses potential harm to both human health and the environment [28]. Furthermore, lead-based perovskites, MAPbX_3_ (X = Cl, Br, I), have comparatively small values of dielectric constants [33]. The fundamental obstacle to the widespread use of solar cell devices is the rate at which charge recombination occurs. This rate reduces with a higher dielectric constant, leading to an enhancement in solar cell efficiency [34]. Finding lead-free and stable substitutes with equivalent optical and thermoelectric capabilities to lead-containing halide perovskites is one way of addressing these environmental issues and meeting the rising need for green energy sources [[35], [36], [37]].

When studying lead-free halide perovskites, researchers often look carefully into several different physical properties, such as structural, optoelectronic and mechanical ones, to better understand the difficulties involved with material applications [[38], [39], [40], [41], [42], [43], [44], [45]]. In order to forecast the potential uses of a material, it is common practice to apply both experimental and theoretical methods of analysis [[46], [47], [48], [49], [50], [51], [52], [53], [54], [55], [56], [57], [58], [59]]. Most recently, there has been substantial research into the use of metal halide perovskite compounds due to their notable optoelectronic characteristics, such as their adaptable transparent bandgap, wide absorption spectrum, narrow emission width, high absorption and charge diffusion, and abundant mobile carriers with a light mass that is efficient [60,61]. Germanium-based, non-toxic, inorganic perovskite compounds, possessing superior conductivity and absorption capabilities compared to Pb-based perovskites, are showing great potential as an alternative to lead (Pb) [33]. Despite its brittleness, CsGeI_3_ has been identified as the most promising Pb-free halide perovskite material, surpassing the ductile CsSnBr_3_, according to previous investigations [62,63].

Researchers have recently focused a lot of attention on the pressure effect on halide perovskites, which is well known to substantially impact the physical and chemical properties of compounds [[64], [65], [66], [67], [68], [69]]. One useful technique involves utilizing hydrostatic pressure or strain engineering for fine-tuning the crystalline stages and electrical band configuration of ABX_3_ materials, thus enhancing comprehension of the consequent shift in the material's rigid bonding [[70], [71], [72]]. However, cation ‘A’ mediates the metal halide interactions without significantly altering the bandgap [73]. Furthermore, octahedral tilting plays a pivotal role in influencing the optical properties, as shown by the correlation between the band gap and lattice constants within the ABX_3_ perovskite structure [74]. Improved optoelectronic and mechanical characteristics were achieved by compressing these lead-free halide perovskites, which resulted in a band gap reduction to zero and the semiconductor-metal transition [[75], [76], [77], [78], [79]]. By extending the orbital overlap integral within tightly correlated electron configurations, pressure can efficiently shorten the distances between atoms and hence improve electron transport. Particularly changes in material characteristics that result in the creation of several new compounds [80]. Consequently, pressure stands as a useful tool for the synthesis of novel compounds [81].

Fang et al. [82], demonstrated a reduction in the band gap of α-CsPbI_3_ perovskites as dopants changed sequentially from Ge to Sn and then to Si, while at a constant doping level, the band gaps diminished with increasing concentrations of doping ions for a given dopant. According to Hossain et al. [83], the inorganic, non-toxic cubic perovskite CsSnCl_3_ shows outstanding optoelectronic properties when the transitions occur to a metal state from a semiconductor state. Based on S.K. Mitro et al.*’s* [84], investigation of the physical properties of Pb-free inorganic cubic perovskites AGeI_3_ (A = Rb, K) at various levels of hydrostatic pressure, the band gaps for both materials contract with pressure, leading to a transition from semiconductor to metal state. According to Safin et al. [85], when the pressure is raised, a noticeable narrowing of the band gap is observed, improving the optical performance and making the compounds more attractive for utilization in solar cell applications. Utilizing the first-principle approach, Hasan et al. [1], carried out an investigation of the structural, electronic, mechanical, and optical properties of FrBX_3_ (B=Ge, Sn; X = Cl, Br, I) perovskite.

As far as we know, no experimental or theoretical research on cubic metal halide FrBX_3_ (B=Ge, Sn; X = Cl, Br, I) compounds under hydrostatic pressure has yet been conducted. Consequently, the goal of this study is to apply first-principles investigations to learn more about the structural, electronic, mechanical, and optical properties of hydrostatically-pressed non-toxic, inorganic compounds FrXCl_3_ (X = Ge and Sn). Our main emphasis has been on investigating how the bandgap and electronic properties of the studied compounds are influenced when subjected to various levels of pressure. Within the applied pressure range, distinct changes in band structure characteristics, as well as variations in absorption, reflectivity, loss function, and elastic behaviors are all examined. The utilization of hydrostatic pressure offers a means to alter the optoelectronic properties of both compounds, making both FrGeCl_3_ and FrSnCl_3_ suitable for applications in solar cells and other optoelectronic devices.

## 2 Computational details

The calculation of ground-state electron energy utilizing Density Functional Theory (DFT) within the Quantum Espresso simulation tool has been taken into consideration for our study [[86], [87], [88]]. We applied the GGA – PBE [89] functional along with the ultrasoft pseudopotential [90], and utilized the Broyden Fletcher-Goldfarb-Shanno (BFGS) [91] minimization process during the geometry optimization process. We employed the kinetic energy cut-offs and charge density cut-offs of 60 Rydberg (Ry) and 600 Rydberg (Ry), respectively, to generate an optimized structure. For the Self-Consistent Field (SCF) and Non-Self-Consistent Field (NSCF) computations, we fix k-points of 8 × 8× 8 and 14× 14× 14, respectively. A maximum force tolerance of below 0.01 eV/Å and 10^−6^ a.u. of convergence threshold was employed in the Self-Consistent Field (SCF) computations. The force convergence threshold was fixed to 10^−3^ a.u. for the ionic minimization in relaxation computations. Once dynamic stability had been established, the perovskite structure's complex dielectric function was determined in order to explore its optical characteristics utilizing the first-order time-dependent perturbation theory [92]. For the purpose of calculating the optical parameters, a gamma-centred mesh of 10 × 10× 10 k-points were employed to sample the Brillouin zone. Subsequently, utilizing an examination of the photon energy spectrum, measured in electron volts (eV), the complex dielectric function's absorption peaks were determined. The complex dielectric function, expressed using the expression, ε(ω) = $\varepsilon_{1}\left(\omega \right)$ + $i\varepsilon_{2}\left(\omega \right)$, is widely acknowledged as being the fundamental equation for determining optical absorption coefficients. The Thermo-PW package [93] was utilized to compute the mechanical properties.

## 3 Results and discussion

### 3.1 Structural properties

**Fig. 1:**
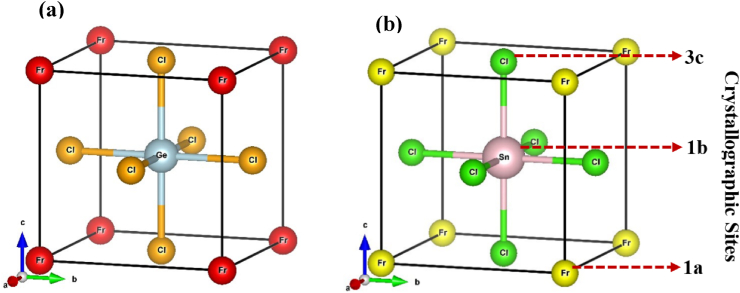
Optimized crystal structure of the inorganic perovskite compounds (a) FrGeCl_3_ and (b) FrSnCl_3_ with crystallographic sites.

**Fig. 2:**
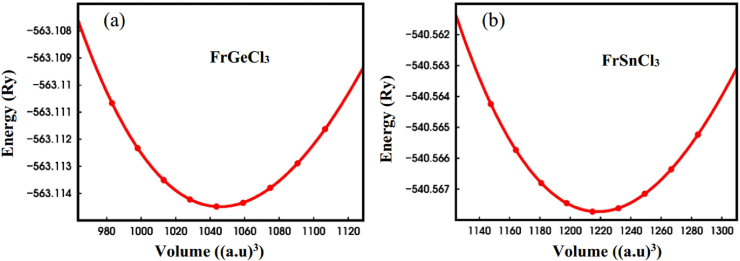
Total energy versus volume of (a) FrGeCl_3_ and (b) FrSnCl_3_ perovskite compounds.

Based on the geometry optimization, FrXCl_3_ (X = Ge and Sn) had space group number 221 (pm $\bar{3}$ m), which crystallized in cubic cells. Fig. 1 shows the optimized unit cell structure along with the crystallographic sites for both compounds, respectively. The Wyckoff coordinates for Fr, X (=Ge, Sn), and Cl are 1a (0, 0, 0), 1b (0.5, 0.5, 0.5), and 3c (0, 0.5, 0.5), respectively, at the corner, body centre, and face centre. The compound's optimized lattice parameters were determined, and the outcomes are represented in Table 1. The Murnaghan equation of state [94] is utilized to determine the energy deviation with respect to volume contrast, shown in Fig. 2.

$$E\left(V\right)=E_{0}+\left[\frac{VB_{0}}{{(B}_{0}^{\prime }-1)B_{0}^{\prime }}\right]\times \left[1-\frac{V_{0}}{V}\right]\;B_{0}^{\prime }+{\left(\frac{V_{0}}{V}\right)}^{B_{0}^{\prime }}-1$$

**Table 1:** Calculated value of lattice constant and unit cell volume of FrXCl_3_ (X = Ge and Sn) perovskites under various hydrostatic pressures.

Compound	Calculated data	Pressure (GPa)					
		0	2	4	6	8	10
**FrGeCl**_**3**_	**a (Å)**	5.37 [This work]	5.25	5.16	5.09	5.03	4.98
		5.37 [1]					
	**V (Å**^**3**^**)**	154.72 [This work]	144.70	137.39	131.87	127.26	123.51
		154.72 [1]					
**FrSnCl**_**3**_	**a (Å)**	5.65 [This work]	5.51	5.40	5.32	5.25	5.19
		5.64 [1]					
	**V (Å**^**3**^**)**	179.41 [This work]	167.28	157.46	150.57	144.70	139.80
		179.83 [1]

**Fig. 3:**
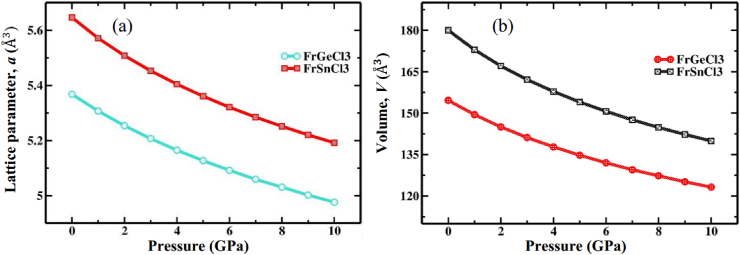
Pressure effect on (a) Lattice parameter and (b) Unit cell volume of halide perovskites of FrGeCl_3_ and FrSnCl_3_.

**Fig. 4:**
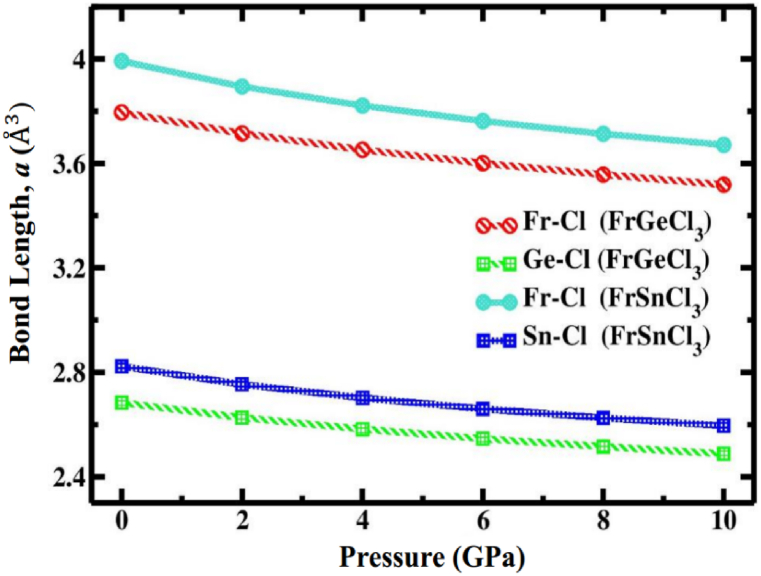
Pressure effect on the bond length of cubic halide perovskites of FrGeCl_3_ and FrSnCl_3_.

For FrGeCl_3_, the minimum total energy value (−563.114 Ry) is located at volume which corresponds to a = 5.37 Å (Table 1), showing no deviation from the reference study (5.64 Å) [1], while for FrSnCl_3_, the minimum total energy value (−540.567 Ry) is located at volume which corresponds to a = 5.65 Å (Table 1), which is comparatively more similar to the previous study (5.64 Å) [1], that ensure high accuracy of this study. According to Table 1, the hydrostatic pressure range of 0–10 GPa was applied to determine the structural properties of both compounds. Fig. 3(a) and b shows the variations in lattice parameter and unit cell volume of FrXCl_3_ (X = Ge and Sn) as the hydrostatic pressure was varied up to 10 GPa, respectively. With increasing pressure levels, there is a drop in both the lattice parameter and the volume of the unit cell, suggesting that interatomic distances are reducing. In Table 2, the values for the bond lengths between the atoms are provided with respect to pressure, as would be predicted, the bond lengths Fr–Cl and X–Cl (X = Ge, Sn) shorten with increasing pressure, as illustrated in Fig. 4. By applying hydrostatic pressure, these bond lengths are slightly worsened, which causes compressive strain to form inside the FrGeCl_3_ and FrSnCl_3_ lattice networks. This implies that the electrical structures of these compounds would be significantly affected by the change in bond length [95].

**Table 2:** Calculated bond lengths in FrXCl_3_ (X = Ge and Sn) perovskites under various hydrostatic pressures.

Pressure (GPa)	Bond length (Å)			
	FrGeCl_3_		FrSnCl_3_	
	Fr–Cl	Ge–Cl	Fr–Cl	Sn–Cl
**0**	3.7955	2.6838	3.9924	2.8231
**2**	3.7149	2.6269	3.8946	2.7539
**4**	3.6522	2.5825	3.8214	2.7022
**6**	3.6006	2.5460	3.7625	2.6605
**8**	3.5574	2.5155	3.7133	2.6257
**10**	3.5187	2.4881	3.6712	2.5959

### 3.2 Electronic properties

**Fig. 5:**
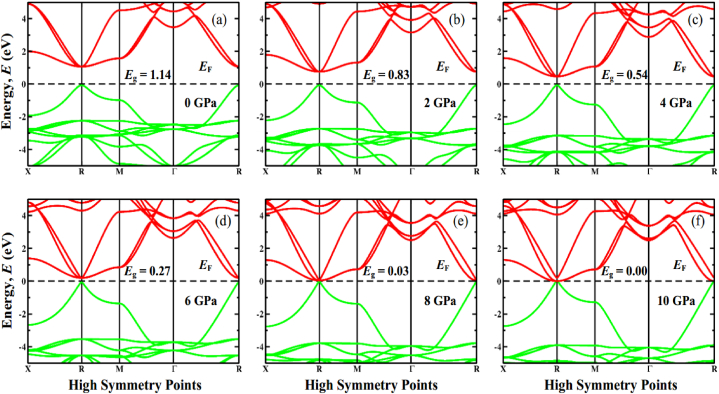
The electronic band structure profile of FrGeCl_3_ under various applied pressure, (a) 0 GPa, (b) 2 GPa, (c) 4 GPa, (d) 6 GPa, (e) 8 GPa, (f) 10 GPa.

**Fig. 6:**
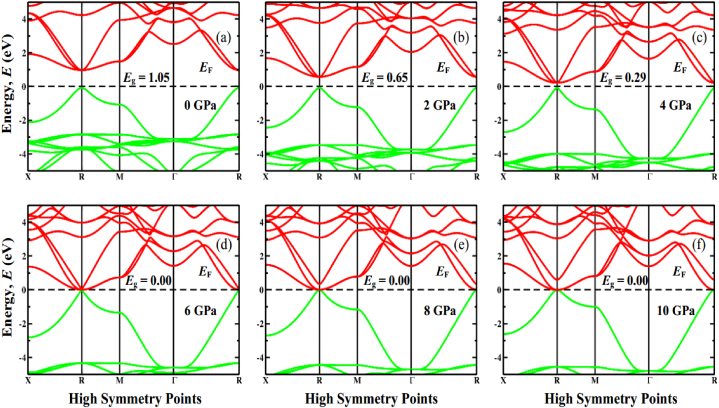
The electronic band structure profile of FrSnCl_3_ under various applied pressure, (a) 0 GPa, (b) 2 GPa, (c) 4 GPa, (d) 6 GPa, (e) 8 GPa, (f) 10 GPa.

In order to get a comprehensive understanding of the optical properties of a material, it is feasible that an understanding of its electronic properties, in particular its band structure and density of states (DOS), will be required. For FrGeCl_3_ and FrSnCl_3_, respectively, under hydrostatic pressure, the band structure layout around the Fermi level has been shown in Fig. 5, Fig. 6. The Fermi level (*E*_F_) is shown via a black horizontal dotted line at 0 eV, and the valence band (VB) and conduction band (CB) are shown via colored lines (green & red) below and above the *E*_F_, respectively. Based on the semi-conductive theory, a material's band structure around the fermi level is a key indicator of the material's physical makeup [96]. Fig. 5 (a) & Fig. 6 (a) represented that when there is no external pressure, the valence band maximum (VBM) and conduction band minimum (CBM) is positioned at the R point without any overlap, ensuring the direct band gap (*E*_g_) nature of both compounds. The direct band gap of FrGeCl_3_ and FrSnCl_3_ is 1.14 eV and 1.05, respectively. The calculated *E*_g_ values for FrXCl_3_ (X = Ge and Sn) are in agreement with those found in previous research [1]. FrGeCl_3_ has an *E*_g_ that is reduced to 0.03 eV when subjected to pressures of 8 GPa. Under increased pressure (10 GPa), the valence band and conduction band meet at the *E*_F_. Similarly, the band gap for FrSnCl_3_ reduces with increasing pressure and disappears at 6 GPa. As a result, each of these investigated compounds undergo a transformation from a semiconducting state to a metallic state under increased pressure, as seen in Fig. 5, Fig. 6. An interaction operates in the reverse direction between the band gap and the external pressure [97]. It might lead to a rise in the attraction across the electron and the ion, which is responsible for a decrease in the lattice constants (Table 1). The band gap in the symmetrical point of the Brillouin zone narrows as the lattice parameter is decreased. Electrons can move from the valence band to the conduction band more readily because of the decrease in *E*_g_. Due to this, absorption of light and conductivity may become more advantageous for utilization in optoelectronics.

**Fig. 7:**
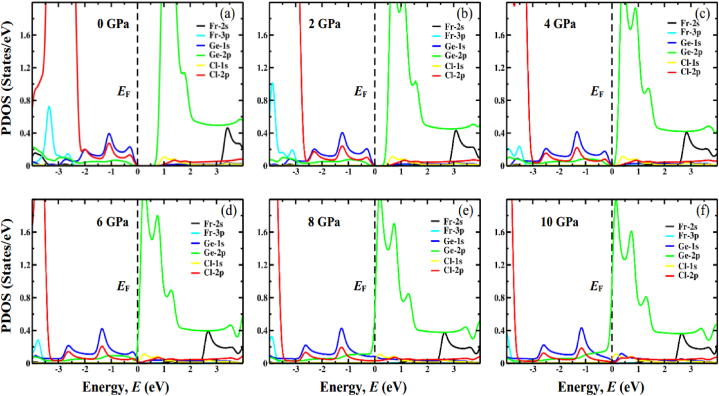
The partial density of states (PDOS) of FrGeCl_3_ under various applied pressure, (a) 0 GPa, (b) 2 GPa, (c) 4 GPa, (d) 6 GPa, (e) 8 GPa, (f) 10 GPa.

**Fig. 8:**
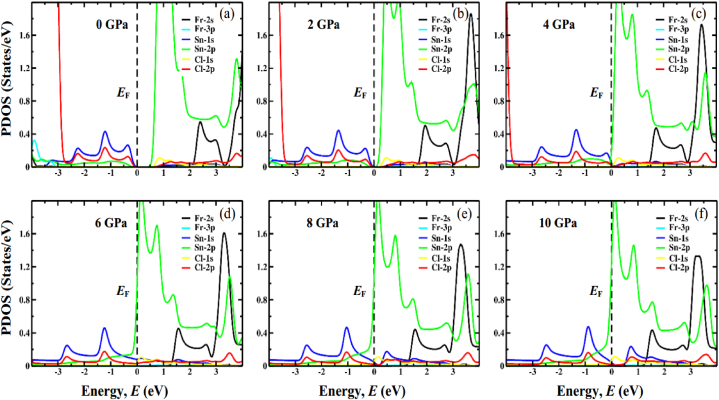
The partial density of states (PDOS) of FrSnCl_3_ under various applied pressure, (a) 0 GPa, (b) 2 GPa, (c) 4 GPa, (d) 6 GPa, (e) 8 GPa, (f) 10 GPa.

To give more insight into the behavior of the electronic system, the computed partial density of states (PDOS) of FrGeCl_3_ and FrSnCl_3_ at various levels of hydrostatic pressure is represented in Fig. 7, Fig. 8, respectively. The *E*_F_ is represented via a vertical black dotted line that passes through 0 eV. Ge-1s (Sn-1s) and Cl-2p orbitals, with a small contribution from Ge-2p (Sn-2p) orbitals, dominate the valence band near the *E*_F_, as seen in Fig. 7, Fig. 8. However, Ge-2p (Sn-2p), Cl-1s and Cl-2p states contribute to the conduction band. It can be demonstrated that the Ge-2p (Sn-2p) orbital plays a key role in lowering the *E*_g_ in both compounds. Increasing external pressure promotes hybridization between Ge-2p (Sn-2p) and Cl-2p by pushing the conduction band nearer to the *E*_F_ and narrowing the band gap. In addition, the pressure-induced narrowing of the Ge(Sn)–Cl bond length (Table 2) may encourage the hybridization between the Ge-2p (Sn-2p) and Cl-2p orbitals in the conduction band. This leads to a lowering of the conduction band minimum (CBM) at the R point of the Brillouin zone and a narrowing of the band gap.

**Fig. 9:**
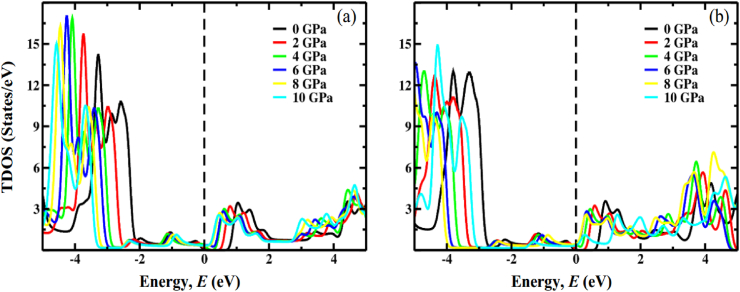
The total density of states (TDOS) of (a) FrGeCl_3_ and, (b) FrSnCl_3_ under various applied pressure.

For both the compounds FrGeCl_3_ and FrSnCl_3,_ total density of states (TDOS) is visualized in Fig. 9, and the observation indicates a progressive shift of distinct band peaks towards the Fermi level (*E*_F_) with increasing applied pressure. These findings confirm the hydrostatic pressure-assured narrowing of the band gap, a characteristic that is also evident in the band structures at the R point.

### 3.3 Mechanical properties

Calculating the elastic variables is necessary in order to assess a material's mechanical characteristics. The response of a crystal to a force can be calculated using the elastic constant C_ij_. Calculating three elastic constants (C_11_, C_12_, and C_44_) thoroughly characterizes the mechanical properties of cubic materials [98]. Three elastic constants (C_11_, C_12_, and C_44_) of FrGeCl_3_ and FrSnCl_3_ were computed using the Thermo_pw algorithm [99]. Table 3 shows the computed values of C_11_, C_12_, C_44_, and Cauchy pressure (C_12_ - C_44_) for FrGeCl_3_ and FrSnCl_3_ under different pressure conditions. For both compounds, the computed elastic constant values at 0 GPa are somewhat closer to the earlier studies [1]. A compound with a cubic crystal structure is said to be mechanically stable when the elastic constants fulfil the Born stability requirements [100]:

$$C_{11}\;–\;C_{12}\;>\;0,C_{44}\;>\;0,C_{11}+2C_{12}\;>\;0$$

**Table 3:** Calculated value of elastic constants *C*_*ij*_ (GPa) in FrXCl_3_ (X = Ge and Sn) perovskites under various hydrostatic pressures.

Pressure (GPa)	Compound	C_11_	C_12_	C_44_	C_12_ - C_44_
0 [1]	FrGeCl_3_	52.41	13.69	12.17	1.52
	FrSnCl_3_	48.41	9.82	6.54	3.28
0	FrGeCl_3_	52.54	13.43	12.18	1.25
	FrSnCl_3_	48.21	9.22	6.49	2.73
2	FrGeCl_3_	69.88	17.62	13.73	3.89
	FrSnCl_3_	66.87	12.87	6.66	6.23
4	FrGeCl_3_	86.40	21.63	15.17	6.46
	FrSnCl_3_	83.94	16.25	6.71	9.54
6	FrGeCl_3_	101.40	25.40	16.51	8.89
	FrSnCl_3_	99.52	19.93	6.61	13.32
8	FrGeCl_3_	114.83	29.29	17.70	11.60
	FrSnCl_3_	115.53	23.78	6.54	17.24
10	FrGeCl_3_	129.70	34.10	18.92	15.17
	FrSnCl_3_	130.90	27.21	6.41	20.80

**Fig. 10:**
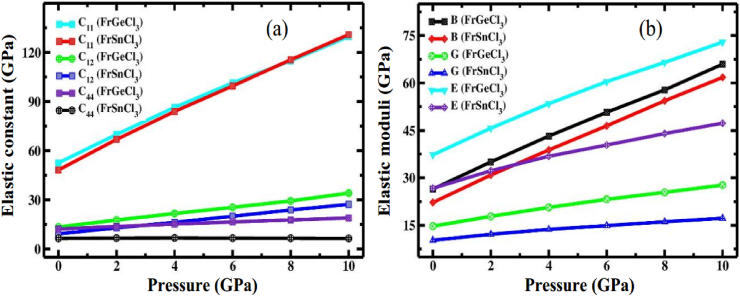
Variation of (a) Elastic constant and (b) Elastic moduli of FrXCl_3_ (X = Ge and Sn) perovskites subjected to different hydrostatic pressures.

Both compounds are stable up to 10 GPa since our predicted elastic constants for both compounds fulfil the stability criteria. Fig. 10(a) illustrates how elastic constants change when studied compounds are subjected to different hydrostatic pressures. The following equations utilize the popular Voigt-Reuss-Hill [[101], [102], [103]] strategy that was used for determining the basic mechanical properties of FrXCl_3_ (X = Ge, Sn), such as the bulk modulus (*B*) and shear modulus (*G*), via computed elastic constants (C_ij_):

$$B=\frac{1}{2}\;\left(B_{R}+B_{V}\right)$$

$$G=\frac{1}{2}\;\left(G_{R}+G_{V}\right)$$

Additionally, the following formulas [[104], [105], [106]] can be utilized to compute Young's modulus (*E*), Poisson's ratio (*v*), and elastic anisotropy (*A*) factor:

$$E=\frac{9BG}{3B+G}$$

$$v=\frac{\left(3B-2G\right)}{2\left(3B+G\right)}$$

$$A=\frac{2C_{44}}{C_{11}-C_{12}}$$

Table 4 demonstrates that as compared to FrSnCl_3_, FrGeCl_3_ has a greater bulk modulus (26.47 GPa). This shows that when compared to FrSnCl_3_, FrGeCl_3_ has the greatest resistance to applied pressure. In terms of shear modulus, FrGeCl_3_ has a higher value (14.74 GPa), followed by FrSnCl_3_ (10.27 GPa), indicating that FrGeCl_3_ has greater shear resistance than FrSnCl_3_. As the value of *E* (Young's Modulus) increases, the material's stiffness also increases; hence FrGeCl_3_ (37.29) will be stiffer than FrSnCl_3_ (26.70). Interatomic distance reduces under pressure, increasing the shear and bulk moduli. Consequently, under high pressure, the mechanical resistance of FrXCl_3_ (X = Ge and Sn) improves significantly. Table 4 shows that *B*, *G*, and *E* values increase under high-pressure conditions. The variation of *B*, *G*, and *E* in response to changes in pressure is depicted in Fig. 10(b).

**Table 4:** The calculated bulk modulus *B* (GPa), shear modulus *G* (GPa), young modulus *E* (GPa), Pugh's ratio *B/G*, Poisson's ratio *ν* and Zener anisotropy *A* of FrXCl_3_ (X = Ge and Sn) perovskites under various hydrostatic pressures.

Pressure (GPa)	Compound	*B*	*G*	*E*	*B/G*	*ν*	*A*
0 [1]	FrGeCl_3_	26.60	14.67	37.18	1.81	0.27	–
	FrSnCl_3_	22.68	10.27	26.77	2.21	0.30	–
0	FrGeCl_3_	26.47	14.74	37.29	1.80	0.26	0.62
	FrSnCl_3_	22.22	10.27	26.70	2.16	0.30	0.33
2	FrGeCl_3_	35.04	17.82	45.71	1.97	0.28	0.52
	FrSnCl_3_	30.88	12.16	32.24	2.54	0.32	0.25
4	FrGeCl_3_	43.22	20.66	53.46	2.09	0.30	0.47
	FrSnCl_3_	38.81	13.72	36.83	2.83	0.34	0.20
6	FrGeCl_3_	50.73	23.22	60.44	2.1849	0.30	0.43
	FrSnCl_3_	46.46	14.90	40.38	3.12	0.35	0.17
8	FrGeCl_3_	57.81	25.42	66.51	2.27	0.31	0.41
	FrSnCl_3_	54.37	16.12	44.00	3.37	0.36	0.14
10	FrGeCl_3_	65.97	27.72	72.93	2.38	0.31	0.40
	FrSnCl_3_	61.78	17.23	47.30	3.58	0.37	0.12

Calculating a structure's anisotropy factor (*A*) provides insight into how stable it is. If A equals 1, the material meets the condition for isotropy. However, if A differs from 1, the material is characterized by anisotropic properties [107]. Both FrGeCl_3_ and FrSnCl_3_ are anisotropic materials, as shown by the values of *A*, which are 0.62 and 0.33, respectively, as displayed in Table 4. Clearly, under hydrostatic pressure, the value of *A* steadily declines from unity, indicating that both FrGeCl_3_ and FrSnCl_3_ perovskites become more anisotropic in nature.

**Fig. 11:**
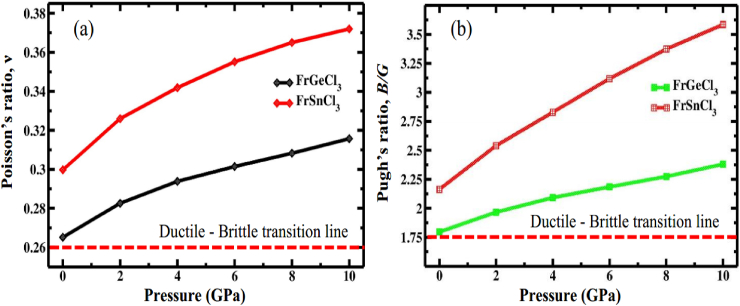
Changes in (a) Poisson's ratio and (b) Pugh's ratio of FrXCl_3_ (X = Ge and Sn) perovskites subjected to different hydrostatic pressures.

There are many factors that help us to determine a compound's brittle or ductile character like (*B/G*) ratio, Poisson's ratio (*ν*) and Cauchy pressure (C_12_ - C_44_). A useful parameter that are taken into consideration to differentiate between brittle and ductile materials is the *B/G* ratio, frequently referred to as Pugh's ratio [82]. Pugh's ratio allows for differentiation between brittle and ductile materials with a value of around 1.75. A material is regarded as ductile (brittle) depending on whether the *B/G* ratio is more (lesser) than 1.75 [108,109]. Both compounds Pugh's ratio was higher than the critical value, emphasizing their ductile nature (Table 4). The critical value for Poisson's ratio is 0.26. If the value of *ν* is below 0.26, a material is considered brittle; if it exceeds 0.26, it is considered ductile [108,109]. Table 4 shows that the Poisson's ratio of both perovskites is larger than 0.26, which demonstrates this perovskite compound's highly ductile nature. From the computed value of *ν* and *B/G* ratio (Table 4), both compounds become more ductile with increasing pressure, as displayed in Fig. 11a and b, respectively.

A material's ductility (positive value) or brittleness (negative value) can be identified using the Cauchy pressure (C_12_–C_44_). As observed in Table 4, both compounds have estimated values of C_11_–C_44_, which are positive for all applied pressures, showing that they are ductile. Both compounds become more ductile as pressure rises due to an increase in the positive C_12_–C_44_ value. Interestingly, the outcome of the data of C_12_ − C_44_ completely resemblances the data of *ν* and *B/G*. However, the computed values of *ν*, *B/G*, and Cauchy pressure (C_12_–C_44_) lead to the following conclusion that FrGeCl_3_ is more ductile than FrSnCl_3_.

### 3.4 Optical properties

To understand how light interacts with a substance, its optical properties must be thoroughly studied [110]. The suitability of a perovskite compound for applications in optoelectronics and photovoltaics can be evaluated by examining its optical properties, which involves analyzing complex dielectric functions: real and imaginary components, absorption coefficient, and electron loss function. In this study, different optical properties of FrXCl_3_ (X = Ge and Sn) perovskites were investigated and shown by altering the lattice parameter using various pressures. The sum of two components determines a material's dielectric function, which is expressed by ε(ω). The first component corresponds to the real part and is shown by the symbol $\varepsilon_{1}\left(\omega \right)$, whereas the second component is the imaginary part and is indicated by the symbol $\varepsilon_{2}\left(\omega \right)$.

$$\varepsilon \left(\omega \right)=\varepsilon_{1}\left(\omega \right)+i\varepsilon_{2}\left(\omega \right)$$

**Fig. 12:**
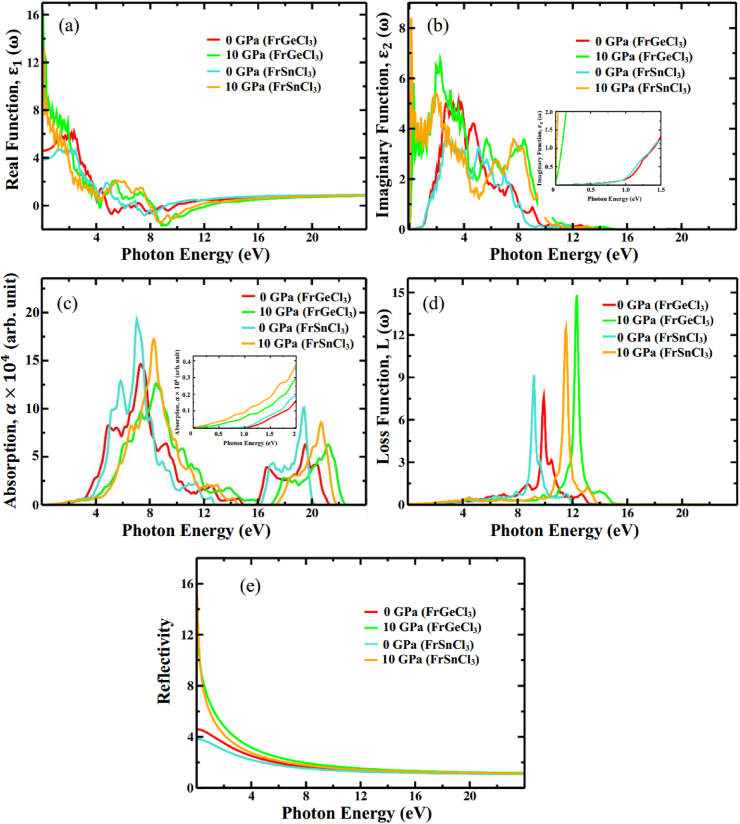
(a) Real portion of dielectric function, (b) Imaginary portion of dielectric function, (c) Absorption (d) Loss function and, (e) Reflectivity of FrGeCl_3_ and FrSnCl_3_ as a function of photon energy under various hydrostatic pressure levels.

The real component of ε(ω) is computed via the Kramers-Kronig transformation, while the imaginary component is computed by assessing the momentum matrix [111,112]. It is possible to examine the polarization and dispersion effects in FrXCl_3_ (X = Ge and Sn) using the real component of the dielectric function. One crucial parameter in the real part is the maximum frequency of zero, which is referred to as the static dielectric function, represented as $\varepsilon_{1}\left(0\right)$. The static dielectric function, $\varepsilon_{1}\left(0\right)$ is an essential parameter that assesses the effectiveness of an optoelectronic device. Most significantly, a higher $\varepsilon_{1}\left(0\right)$ value suggests a greater possibility of applications in optoelectronic and photovoltaic devices due to lower recombination of charges [113]. As shown in Fig. 12(a), the calculated value of $\varepsilon_{1}\left(0\right)$ for FrGeCl_3_ is 4.73 at 0 GPa, whereas 16.72 at 10 GPa. In a similar way, for the FrSnCl_3_ perovskite, the value of $\varepsilon_{1}\left(0\right)$ at high pressure (10 GPa) is larger (∼13.24) than the value (∼3.94) at 0 GPa. Notably, As subjected to 10 GPa pressure, $\varepsilon_{1}\left(0\right)$ for both perovskite materials increase significantly due to a lower rate of charge recombination, making these materials more preferable for optoelectronic applications. The subsequent recombination rate will be reduced as a result of the decrease in *E*_g_ values, which guarantees that a higher number of electrons can migrate from valence bands to conduction bands. Consequently, with an increase in applied pressure, both FrGeCl_3_ and FrSnCl_3_ exhibit increased static dielectric values at zero photon energy. Also, according to findings, the reduction in bandgap under pressure causes both halide perovskites to have a larger dielectric constant peak and experience a shift towards lower photon energy (resulting in redshift). This result suggests that the perovskite material FrGeCl_3_ is more suited to device application than FrSnCl_3_. The Penn model [58] suggests that the band gap of a perovskite material exhibits an inverse relationship with $\varepsilon_{1}\left(0\right)$. This characteristic can be explained by the following equation:

$$\varepsilon_{1}\left(0\right)\approx 1+{\left(\frac{{\hbar \omega }_{p}}{E_{g}}\right)}^{2}$$

Consequently, the results obtained for the electronic properties (Fig. 5, Fig. 6, Fig. 7, Fig. 8, Fig. 9) consistently conform to this model.

Understanding light absorption and the energy-storing capacity of the crystal structure brought on by unbiased charge excitations requires knowledge of the imaginary part of ε(ω) [114]. Information on the energy of inter-band transitions that are close to the *E*_F_ and the electronic bandgap can be obtained by the analysis of imaginary dielectric function $\varepsilon_{2}\left(\omega \right)$, that can be employed in assessing the optical characteristics of a material, including its reflectivity and absorption [115]. At 0 GPa, the $\varepsilon_{2}\left(\omega \right)$ value is higher in the visible and early ultraviolet regions, signifying a high level of absorption in those spectral ranges [116]. The highest peaks in $\varepsilon_{2}\left(\omega \right)$ for FrGeCl_3_ (FrSnCl_3_) at zero pressure occurred at an optical position of 5.45 (3.76), suggesting that the absorbed photon's energy was approximately 3.98 eV (2.85), as displayed in Fig. 12(b). When subjected to high pressure, the imaginary section of the dielectric functions for both halide perovskite grows and moves toward lower energy areas. The imaginary absorption peaks regulate the transfer of carriers from the VBs to the CBs. The bandgap and lattice constant variations are held accountable for the peak's displacement [115]. The higher values of $\varepsilon_{1}\left(\omega \right)$ and $\varepsilon_{2}\left(\omega \right)$ at lower energies, coupled with lower values of $\varepsilon_{1}\left(\omega \right)$ and $\varepsilon_{2}\left(\omega \right)$ at higher energies, highlight the potential suitability of both compounds in microelectronics and integrated circuits.

The optical absorption coefficient (α) signifies the energy absorbed by a material per unit length [85] and provides an understanding of the material's efficiency in converting solar energy optimally. Under various pressure scenarios, the absorption coefficient of FrGeCl_3_ and FrSnCl_3_ in terms of photon energy is displayed in Fig. 12(c). Because of the presence of a band gap in both FrGeCl_3_ and FrSnCl_3_ at ambient pressure, the absorption does not begin at zero photon energy. However, both compounds exhibit more prominent peaks in the UV range, indicating that these materials function well as absorbers in this region at 0 GPa. As the pressure is increased, there is a noticeable shift of the absorption edge towards the higher energy area of the spectrum. Notably, the absorption spectra initiate from nearly 0 eV at 10 GPa. Furthermore, the absorption in the visible area is significantly greater at 10 GPa compared to what is observed at zero pressure. This suggests that both FrGeCl_3_ and FrSnCl_3_ have the capacity to utilize visible light energy for photovoltaic conversion under high-pressure conditions, which could potentially augment solar cell's efficiency.

The “electron loss function,” denoted by L(ω), calculates how much energy electrons lose while moving across a dielectric substrate [117]. The electron loss function, L(ω), can be computed utilizing the expression shown below.

$$L\left(\omega \right)=j\;\left(-\frac{1}{\varepsilon \left(\omega \right)}\right)$$

The energy loss happens when photons with energies surpassing the bandgap of materials are emitted. The pressure-dependent electron loss function (ELF) for FrGeCl_3_ and FrSnCl_3_ is shown in Fig. 12 (d). The L(ω) for FrGeCl_3_ and FrSnCl_3_ at zero pressure increases in the energy range of ∼8–14 eV to ∼7 to 13, respectively. According to the findings shown in Fig. 12 (d), there was no dispersion below the bandgap energy for both FrGeCl_3_ and FrSnCl_3_. When subjected to high pressure, a significant change to decreased photon energy is observed via optical loss (redshift) for both compounds. Our compounds become more responsive and efficient in absorbing light within the infrared and visible spectrums due to the lower influence of energy loss below 4.5 eV.

In optoelectronic and solar cell applications, reflectivity stands as a crucial optical property which analyses the surface nature of materials [118]. The pressure-dependent reflectivity of FrGeCl_3_ and FrSnCl_3_ are depicted in Fig. 12(e). These plots showed that the reactivity of both compounds was relatively low at zero pressure. Maximum reflectivity for both compounds is attained at 0 eV photon energy. The implementation of outside pressure has shown little impact on the reflectivity pattern in the high-energy region. As hydrostatic pressure rises, the reflectivity of FrGeCl_3_ and FrSnCl_3_ in the visible range also increases, rendering them promising candidates for utilization in solar cells and optoelectronic applications.

## 4 Conclusion

Under hydrostatic pressure, the simple cubic perovskites material FrXCl_3_ (X = Ge and Sn) is explored for its structural, electronic, optical, and mechanical properties utilizing the DFT approach. The lattice constant and cell volume closely align with earlier research, and they exhibit a tendency to decrease when pressure is applied. Increasing pressure resulted in a pronounced attractive interaction between the valence band and the conduction band, which contributed to CB and VB overlap ultimately, both studied semiconductors were transformed into conductors after a certain level of pressure was applied. Both FrGeCl_3_ and FrSnCl_3_ compounds exhibit a growing affinity for ductility with increasing pressure, according to investigations on Pugh's ratio and Poisson's ratio, and the material exhibits promise for applications where practical devices need to be highly ductile. For optoelectronic applications, compared to zero pressure systems, pressure-sensitive FrGeCl_3_ and FrSnCl_3_ perovskites are more likely to be utilized, according to the varied optical characteristics. Both examined materials displayed a red-shifted behavior in terms of their electronic and optical band gaps. Depending on the direct energy gap falling within the range of solar cells, the reported properties make our studied compounds extremely essential for optoelectronic applications.

## Data availability

The data that support the findings of this study are available from the corresponding author upon reasonable request.

## CRediT authorship contribution statement

**Asif Hosen:** Writing – original draft, Methodology, Formal analysis, Data curation, Conceptualization. **Md. Rasidul Islam:** Writing – review & editing, Writing – original draft, Validation, Supervision, Software, Resources, Methodology, Investigation, Formal analysis, Conceptualization. **Shahriar Haque Badhan:** Writing – original draft, Visualization, Investigation, Formal analysis, Data curation.

## Declaration of competing interest

The authors declare that they have no known competing financial interests or personal relationships that could have appeared to influence the work reported in this paper.
